# Targeting Interferon-Gamma (IFN-γ)-Related Signalling Pathways in Inflammatory Bowel Disease: Emerging Inhibitors and Therapeutic Advances

**DOI:** 10.1155/mi/3181200

**Published:** 2025-10-21

**Authors:** Md. Mizanur Rahaman, Phurpa Wangchuk, Subir Sarker

**Affiliations:** ^1^Biomedical Sciences and Molecular Biology, College of Medicine and Dentistry, James Cook University, Townsville, Queensland 4811, Australia; ^2^Australian Institute of Tropical Health and Medicine, James Cook University, Townsville, Queensland 4811, Australia; ^3^College of Science and Engineering, James Cook University, Nguma Bada Campus, McGregor Road, Smithfield, Cairns, Queensland 4878, Australia; ^4^Australian Institute of Tropical Health and Medicine, James Cook University, Nguma Bada Campus, McGregor Road, Smithfield, Cairns, Queensland 4878, Australia; ^5^Department of Microbiology, Anatomy, Physiology and Pharmacology, School of Agriculture, Biomedicine and Environment, La Trobe University, Melbourne, Victoria 3086, Australia

**Keywords:** cytokines, IBD, IFN-γ, inflammation, inhibitors

## Abstract

Inflammatory bowel disease (IBD) encompasses a group of chronic and relapsing inflammatory disorders of the gastrointestinal tract, driven by a multifaceted interplay between genetic predisposition, environmental factors and dysregulated immune responses. Central to its immunopathogenesis is the aberrant activation of pro-inflammatory cytokine networks, among which interferon-gamma (IFN-γ) has been increasingly recognised as a critical mediator of mucosal damage and disease perpetuation. IFN-γ exerts pleiotropic effects on both innate and adaptive immune compartments, orchestrating a pathogenic immune milieu that disrupts intestinal epithelial integrity and sustains chronic inflammation. Recent therapeutic advances have focused on the modulation of IFN-γ signalling as a targeted approach to restoring intestinal homeostasis. A growing repertoire of IFN-γ inhibitors—including neutralising monoclonal antibodies (MAbs), small-molecule Janus kinase (JAK) inhibitors and bioactive phytochemicals—are being explored for their capacity to attenuate IFN-γ-driven inflammatory cascades. These agents offer distinct mechanistic profiles, targeting various nodes of the IFN-γ axis, and hold significant promise for addressing therapeutic gaps in refractory IBD. This review provides a comprehensive evaluation of emerging IFN-γ-targeted therapies, detailing their mechanisms of action, preclinical and clinical efficacy and translational potential. By elucidating the therapeutic landscape of IFN-γ modulation, this study aims to inform the development of more effective and personalised treatment strategies for patients with IBD.

## 1. Introduction

Inflammatory bowel disease (IBD) is a chronic inflammatory disease of the gastrointestinal tract, primarily divided into two principal subtypes such as ulcerative colitis (UC) and Crohn's disease (CD) [1]. CD is characterised by transmural inflammation that can affect any region of the gastrointestinal tract, most commonly the terminal ileum or perianal region, in a discontinuous manner [2]. Unlike UC, CD is frequently associated with a spectrum of complications such as abscesses, fistulas and strictures. UC; however, is limited to the colonic mucosa and is marked by mucosal inflammation, predominantly impacting the rectum and spreading proximally in a continuous manner [3]. The clinical presentation of IBD often encompasses non-specific gastrointestinal symptoms such as abdominal pain, persistent diarrhoea and rectal bleeding [4]. As highlighted by the World Health Organization, IBD remains among the most challenging chronic diseases globally, with rising incidence and mortality rates—particularly in newly industrialised countries—over the past five decades [5–7]. In addition to physical symptoms, IBD is often associated with psychological comorbidities and malnutrition, contributing to increased healthcare costs and significantly impaired quality of life for affected individuals [8]. Although the precise aetiology of IBD remains largely unknown, emerging evidence suggests that genetic predisposition, environmental factors, immune responses and intestinal microbiota are both functionally interconnected in its pathogenesis [9–11]. Historically, much of the investigative focus has centred on characterising the role of immune cell populations in the initiation and progression of IBD [12].

Building upon foundational insights into the immunopathogenesis of IBD, contemporary immunological and genetic investigations have increasingly underscored the pivotal role of cytokines as central modulators of intestinal inflammation [13, 14]. Among these, interferon-gamma (IFN-γ), a prototypical pro-inflammatory cytokine, has garnered significant attention due to its multifaceted involvement in IBD pathophysiology. IFN-γ orchestrates key immune responses by directing T helper cell differentiation, activating macrophages and enhancing the expression of major histocompatibility complex (MHC) class I and II molecules—thereby amplifying antigen presentation and perpetuating mucosal immune activation [15, 16]. Notably, elevated mucosal levels of IFN-γ have been consistently observed in patients with CD, and its critical role in mediating intestinal inflammation has been substantiated through various experimental colitis models [17].

At the mechanistic level, IFN-γ has been shown to disrupt epithelial barrier function by down-regulating tight junction (TJ) proteins, including zonula occludens (ZO)-1 and occludin, thereby facilitating paracellular permeability and translocation of luminal antigens [18–20]. Furthermore, emerging evidence indicates that IFN-γ also disrupts vascular barrier function by impairing adherens junction integrity through the degradation of VE-cadherin, further exacerbating mucosal immune infiltration and inflammatory burden [21].

Given its central pathogenic role, IFN-γ has emerged as a compelling therapeutic target for modulating intestinal inflammation in IBD. Therapeutic strategies aimed at inhibiting IFN-γ signalling are gaining momentum, particularly in cases where patients exhibit suboptimal responses to conventional therapies. Current research is focused on the discovery and characterisation of effective IFN-γ antagonists capable of attenuating mucosal inflammation and promoting epithelial homeostasis. Promising avenues include the application of monoclonal antibodies (MAbs) targeting IFN-γ or its receptor, the development of receptor antagonists and the exploration of bioactive phytochemicals with immunomodulatory properties [22–27]. This review seeks to comprehensively examine the therapeutic potential of IFN-γ inhibitors in the context of IBD, with particular emphasis on their underlying mechanisms of action and translational relevance.

## 2. Materials and Methods

This study adhered to stringent selection criteria to ensure the reliability and relevance of collected data. We sourced literature from high-impact and widely recognised scientific databases, including PubMed, Web of Science, Scopus and Google Scholar, prioritising peer-reviewed studies. The selection process was guided by specific keywords and MeSH terms, such as IFN-γ, IBD, IFN-γ inhibitors, MAbs, IFN-γ receptor (IFNGR) blockers, plant-derived compounds, pharmacological activity, molecular mechanisms and therapeutic effects. We included studies published up to January 2025, ensuring the inclusion of the most recent advancements in the field. Articles were further screened based on study design, sample size and experimental validity. Studies published in predatory journals, non-English publications, conference abstracts, preprints lacking peer review and those that did not align with our research objectives were excluded to maintain the integrity and scientific rigour of our review.

## 3. Results and Discussion

### 3.1. Role of IFN-γ in the Pathogenesis of IBD

**Figure 1:**
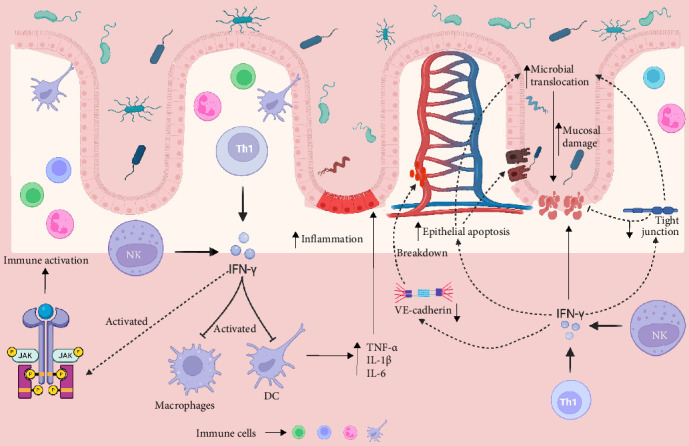
Schematic representation of IFN-γ–mediated immunopathogenic mechanisms in IBD. This figure delineates the complex immunological cascade underlying IBD pathogenesis, with a central focus on the pivotal role of IFN-γ. IFN-γ, predominantly secreted by (Th1) lymphocytes and natural killer (NK) cells, orchestrates a pro-inflammatory response through the activation of macrophages and dendritic cells (DCs), thereby amplifying mucosal inflammation. Activated antigen-presenting cells subsequently produce a repertoire of pro-inflammatory cytokines, including TNF-α, IL-1β and IL-6, which synergistically perpetuate inflammatory signalling. Additionally, IFN-γ induces epithelial barrier dysfunction and tissue injury via activation of the Janus kinase–signal transducer and activator of transcription (JAK–STAT) signalling pathway, linking cytokine-mediated immune activation to sustained epithelial damage and compromised barrier integrity.

IFN-γ is a potent pro-inflammatory cytokine primarily secreted by T-helper 1 (Th1) lypmholcytes and natural killer (NK) cells, plays a pivotal role in the immunopathogenesis of IBD, with particular prominence in CD [28–30]. Acting as a key immunomodulatory molecule, IFN-γ exerts its effects by activating macrophages and dendritic cells (DCs), which in turn enhances the secretion of key inflammatory mediators such as tumour necrosis factor-alpha (TNF-α), interleukin-1β (IL-1β) and interleukin-6 (IL-6), thereby perpetuating chronic intestinal inflammation [31–34]. Beyond its role in immune cell activation, IFN-γ critically impairs intestinal epithelial barrier function through multiple mechanisms. It downregulates the expression of TJ proteins (e.g., claudins and occludins), induces epithelial cell apoptosis and facilitates translocation of antigens and luminal microorganisms throughout the compromised barrier, contributing to mucosal damage and immune activation [35–37]. Furthermore, IFN-γ also activates the Janus kinase (JAK)-signal transducer and activator of transcription (STAT) signalling pathway, promoting the transcription of pro-inflammatory genes and perpetuating aberrant immune responses that underpin the chronicity and relapsing nature of IBD [38, 39]. In addition, IFN-γ contributes to disease pathogenesis by disrupting vascular integrity, particularly through the disassembly of adherens junctions via VE-cadherin destabilisation, thus promoting leukocyte transmigration and enhancing intestinal inflammation [21]. Collectively, the multifaceted pathogenic mechanisms driven by IFN-γ underscore its critical contribution to the initiation and perpetuation of IBD. A schematic representation of IFN-γ-mediated mechanisms in IBD pathogenesis is illustrated in Figure 1.

### 3.2. IFN-γ Inhibitors for IBD Treatment

**Figure 2:**
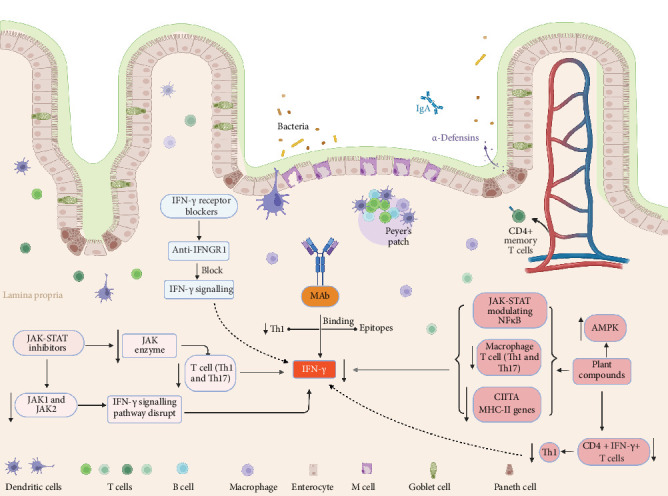
Schematic representation of IFN-γ signalling inhibitors in the intestinal immune system and its therapeutic targeting in inflammatory bowel disease (IBD). This figure illustrates the role of IFN-γ in intestinal immune regulation, highlighting its impact on epithelial barrier function, immune cell activation and potential therapeutic interventions. IFN-γ, secreted primarily by Th1 cells, interacts with its receptor, triggering JAK-STAT signalling, which subsequently regulates immune responses through macrophage activation and MHC-II gene expression. Therapeutic strategies, including JAK-STAT inhibitors, IFN-γ receptor blockers (e.g., Anti-IGNGR1) and monoclonal antibodies (MAbs), are shown as potential approaches to mitigate IFN-γ-mediated inflammation. Additionally, plant-derived compounds modulating AMPK pathways are depicted as alternative therapeutic agents influencing CD4+ IFN-γ+ T cell activity.

Targeting IFN-γ signalling has emerged as a promising therapeutic strategy to mitigate disease progression. Several biologics and small-molecule inhibitors have been developed to neutralise IFN-γ or block its downstream signalling pathways. Preclinical studies and early-phase clinical trials have demonstrated the potential of IFN-γ inhibitors in reducing inflammation, restoring immune homeostasis and alleviating disease symptoms are summarised below with a mechanistic insight in Figure 2.

#### 3.2.1. Direct IFN-γ Inhibitors

##### 3.2.1.1. MAbs Targeting IFN-γ

MAbs targeting IFN-γ represent a promising therapeutic avenue and strategically targeted immunotherapeutic modality within the expanding armamentarium for the management of IBD (summarised in Table 1) [44]. These antibodies exert their therapeutic effects by selectively binding to specific epitopes on the IFN-γ molecule, thereby sterically hindering its interaction with cognate surface receptors on immune effector cells. This blockade effectively abrogates the activation of downstream JAK–STAT signalling pathways, culminating in the suppression of IFN-γ-mediated pro-inflammatory transcriptional programmes [24, 25]. In the context of IBD particularly CD and UC, two MAbs have been investigated so far for their potential ability to mitigate disease severity through IFN-γ inhibition.

The immunoneutralization of IFN-γ is particularly relevant given its well-established role in driving Th1-type immune responses, which are critically implicated in the pathogenesis of both CD and UC. Within this context, two MAbs have undergone pre-clinical and/or clinical investigation, aiming to attenuate disease activity and mucosal inflammation through precise modulation of IFN-γ bioavailability and signalling (Table 1). These agents underscore a paradigm shift towards cytokine-specific immune intervention, with the potential to refine disease management strategies and minimise systemic immunosuppression.

*3.2.1.1.1*. *Fontolizumab*. Fontolizumab is a humanised IgG1 MAb that selectively targets IFN-γ, a key cytokine involved in Th1-driven immune responses [40, 45]. Given the central role of IFN-γ in the pathogenesis of CD [21], fontolizumab was developed as a potential therapeutic intervention to mitigate intestinal inflammation through targeted cytokine blockade [40]. By binding to endogenous human IFN-γ, fontolizumab inhibits the expression of IFN-γ–responsive genes that are characteristically upregulated in CD [46, 47]. Initial clinical trials reported modest reductions in inflammatory biomarkers and symptomatic improvement in subsets of patients with moderate-to-severe CD [48]. However, subsequent trials failed to demonstrate sustained clinical efficacy, and the development of fontolizumab for IBD was ultimately discontinued [49]. These findings underscore the complexity of IFN-γ mediated inflammation in IBD and suggest that broader immune modulation may be required for effective disease control.

*3.2.1.1.2*. *Emapalumab*. Emapalumab is a fully human IgG1 MAb that neutralises IFN-γ by binding directly to the cytokine and preventing its interaction with cell surface receptors [42]. It is currently FDA-approved treatment for primary hemophagocytic lymphohistiocytosis (HLH) in the United States, a severe hyperinflammatory disorder characterised by dysregulated IFN-γ signalling [50]. By specifically targeting IFN-γ, emapalumab inhibits its biological activity and mitigates the cytokine-driven inflammatory cascade, including the prevention of cytokine storms [42]. While this antibody not yet approved for IBD, its mechanism suggests potential therapeutic applications in refractory IBD cases where IFN-γ plays a dominant pathogenic role [21]. The clinical success of emapalumab in HLH supports further exploration of IFN-γ blockade in context of chronic intestinal inflammation, offering a rationale for its investigation in IBD.

##### 3.2.1.2. IFNGR Blockers

IFNGR blockers, also referred to as IFNGR antagonists, represent a class of immunomodulatory agents designed to inhibit the IFN-γ-mediated signalling cascade [22]. The biological activity of IFN-γ is mediated through its binding to the heterodimeric IFNGR, which comprises two distinct subunits: IFNGR1 and IFNGR2 [16]. Ligand engagement with this receptor complex triggers activation of the JAK–STAT pathway, subsequently inducing the transcription of a spectrum of pro-inflammatory genes implicated in the pathogenesis of intestinal inflammation [16, 51, 52].

Targeted blockade of the IFNGR1 subunit using MAbs has been proposed as a potential therapeutic strategy to attenuate IFN-γ-driven inflammation, thereby offering a novel avenue for immune modulation in IBD [23]. By disrupting IFNGR engagement, these agents may effectively suppress downstream inflammatory signalling. However, given the pleiotropic roles of IFN-γ in host immune defence, particularly in antimicrobial responses, the broad immunosuppressive potential of IFNGR inhibitors necessitates careful evaluation. The associated risk of opportunistic infections remains a significant concern, and to date, there is a paucity of clinical studies investigating the safety, efficacy, and long-term consequences of anti-IFNGR1 therapy in IBD. This underscores a critical gap in current research and highlights the need for rigorous preclinical and clinical investigations to elucidate the therapeutic viability of IFNGR blockade in the context of chronic intestinal inflammation.

#### 3.2.2. Indirect Modulation of IFN-γ Pathways in IBD

##### 3.2.2.1. JAK–STAT Inhibitors

JAK–STAT inhibitors (summarised in Table 2 and Figure 2) represent a promising class of therapeutics for the treatment of IBD. These small-molecule drugs act by disrupting the JAK–STAT signalling cascade, a critical intracellular pathway involved in the transduction of pro-inflammatory cytokine signals, including IFN-γ-a key mediator in IBD pathogenesis [65–68]. By blocking this pathway, JAK inhibitors can suppress the production and activity of IFN-γ, thereby attenuating intestinal inflammation and ameliorating clinical symptoms [59, 68, 69]. Several JAK inhibitors have received regulatory approval for the treatment of moderate-to-severe IBD, particularly UC, owing to their capacity to simultaneously inhibit multiple cytokine signals implicated in disease progression, including IFN-γ [70–72]. Their broad-spectrum immunomodulatory effects position them as a key therapeutic option in evolving IBD treatment paradigm.

*3.2.2.1.1*. *Tofacitinib*. Tofacitinib, an oral JAK inhibitor, has emerged as a pivotal agent in modulating inflammatory responses in IBD [73, 74]. By selectively targeting JAK enzymes, tofacitinib disrupts the intracellular signalling cascades initiated by cytokines such as IFN-γ, thereby inhibiting downstream effects including T cell specifically Th1 and Th17 activation and pro-inflammatory cytokine production [12, 73]. Clinical studies have demonstrated that tofacitinib treatment leads to significant reductions in IFN-γ levels in patients with IBD, correlating with symptomatic improvement and induction of clinical remission [75]. These findings support its role as an effective therapeutic option, particularly in cases where IFN-γ driven immune activation plays a dominant pathogenic role.

*3.2.2.1.2*. *Upadacitinib.* Upadacitinib, a newly registered JAK inhibitor targeted for JAK1, could be a potential choice in people with UC [76]. By specifically inhibiting JAK1, upadacitinib disrupts the JAK–STAT signalling cascade, thereby preventing the phosphorylation and activation of downstream transcription factors and suppressing the activity of multiple pro-inflammatory cytokines, including IFN-γ [54]. Inhibition of IFN-γ signalling contributes to the attenuation of intestinal inflammation, positioning upadacitinib as a promising therapeutic candidate for IBD management [55]. Notably, its clinical efficacy and safety have been validated in phase 3 trials (NCT02819635 and NCT03653026), demonstrating significant improvements in patients with moderately to severely active UC [56].

*3.2.2.1.3*. *Filgotinib.* Filgotinib is an orally administered, selective JAK1 inhibitor that targets a critical component of the JAK–STAT signalling cascade [65, 77]. This pathway is instrumental in mediating the biological effects of several pro-inflammatory cytokines including IFN-γ, which are involved in the inflammatory response in the gut [51]. By selectively inhibiting JAK1, filgotinib effectively suppresses IFN-γ signalling, resulting in reduced circulating levels of this cytokine [57]. Clinical studies have demonstrated that filgotinib treatment leads to significant decreases in IFN-γ and other inflammatory biomarkers, which are associated with improvements in clinical disease activity indices and endoscopic outcomes in patients with UC [78, 79]. These findings support filgotinib's potential as a targeted therapy for patients with moderate-to-severe UC.

*3.2.2.1.4*. *Ruxolitinib*. Ruxolitinib is a JAK inhibitor, specifically targeting JAK1 and JAK2, which are essential components of the JAK–STAT pathway [80, 81]. By inhibiting JAK1 and JAK2, ruxolitinib can disrupt the IFN-γ signalling pathway, reducing the production of pro-inflammatory cytokines and mitigating inflammation [59]. Preclinical studies have demonstrated that ruxolitinib can reduce inflammation in animal models of IBD, and it has also been used in clinical trials for patients with IBD, showing promising results, indicating its potential as a therapeutic candidate for managing chronic intestinal inflammation [60, 61].

*3.2.2.1.5*. *Baricitinib.* Baricitinib is a JAK inhibitor, specifically targeting JAK2/STAT3 signalling pathways [62]. This pathway plays a critical role in development of inflammatory diseases by regulating T cell differentiation and proliferation, as well as cytokine secretion [51, 82]. A study by Wu et al. [62] demonstrated that baricitinib effectively inhibits JAK2/STAT3 signalling, leading to suppression of IFN-γ and other pro-inflammatory cytokines, including IL-6 and IL-17A. These findings suggest that baricitinib could be a valuable therapeutic strategy for targeting immune dysregulation in IBD.

*3.2.2.1.6*. *Peficitinib.* Peficitinib is an oral inhibitor of JAK1, JAK2, JAK3, and Tyk2 (pan-JAK) that has demonstrated promising results in the treatment of IBD [56]. By inhibiting these JAK enzymes, peficitinib disrupts the signalling of multiple pro-inflammatory cytokines, including IFN-γ, a key mediator in IBD pathogenesis [83]. Clinical studies have highlighted the potential of peficitinib, particularly in UC, where it has shown efficacy in achieving mucosal restore, remission and clinical response in individuals with moderate-to-severe infection, as demonstrated in a phase 2b trial [56, 63, 84, 85]. However, no ongoing trials are presently registered to continue peficitinib evaluation in UC or CD patients [85].

##### 3.2.2.2. Plant-Based IFN-γ Inhibitors

Plant compounds have been utilised for centuries due to their numerous health benefits, with substantial evidence supporting their anti-inflammatory properties. Recent research has identified several plant-derived compounds with the ability to inhibit production of pro-inflammatory cytokines IFN-γ, a key mediator in inflammatory responses [86, 87]. Some of these bioactive compounds, as listed in Table 3, have demonstrated potential in suppressing IFN-γ production and modulating various inflammatory pathways described in Figure 2, highlighting their therapeutic promise in the management of inflammatory disorders. However, plant-derived compounds such as curcumin, resveratrol and epigallocatechin gallate (EGCG) have demonstrated IFN-γ inhibitory effects in preclinical IBD models, although no dedicated clinical trials have specifically evaluated their efficacy as IFN-γ inhibitors in IBD [89, 96, 97]. The lack of trials may be attributed to challenges including poor bioavailability, pharmacokinetic variability, regulatory hurdles and the multifactorial mechanisms of these phytochemicals that extend beyond IFN-γ modulation. Nonetheless, a few small-scale clinical studies have examined curcumin and EGCG in IBD, but these did not specifically focus on IFN-γ as a primary endpoint [98–101]. Collectively, this highlights the need for well-designed, mechanistic trials with cytokine-specific endpoints to validate the translational potential of plant-based IFN-γ inhibitors in IBD.

*3.2.2.2.1*. *Curcumin (Curcuma longa).* Curcumin belongs to the family of natural compounds collectively called curcuminoids and it possesses remarkable anti-inflammatory properties particularly, suggested as a remedy for digestive diseases such as IBD [102]. Among its key mechanisms, curcumin has been identified as a potent inhibitor of IFN-γ, a pro-inflammatory cytokine implicated in the pathogenesis of IBD [96, 103]. IFN-γ plays a pivotal role in disrupting intestinal homeostasis by activating macrophages [104, 105], modulating T cell activity [98] and promoting epithelial barrier dysfunction [21]. Curcumin mitigates these effects by downregulating IFN-γ expression, modulating JAK–STAT signalling and inhibiting NF-κB activation, thereby attenuating inflammatory cascades within the gut [106–109]. Additionally, curcumin suppressed the transcription of IFN-γ-induced genes, such as CII-TA, MHC-II genes (HLA-DRα, HLA-DPα1 and HLA-DRβ1) and T cell chemokines (CXCL9,10) [96].

*3.2.2.2.2*. *Resveratrol (Grapes, Berries and Peanuts).* Resveratrol, a naturally occurring polyphenol, has shown promise in preclinical studies for its anti-inflammatory and antioxidant properties, which are crucial in managing IBD [110, 111]. Notably, resveratrol treatment has been associated with a decrease in IFN-γ expression in murine models of colitis [89]. For instance, resveratrol administration significantly reduced the number of CD3+ T-cell infiltrates expressing IFN-γ in the lamina propria and mesenteric lymph nodes of mice with colitis caused by dextran sulphate sodium (DSS) [89]. Additionally, resveratrol treatment led to a decrease in CD4+IFN-γ+ T cells in colitis mice, suggesting a suppression of Th1-mediated immune responses [112].

*3.2.2.2.3*. *EGCG (Green Tea).* EGCG, a primary catechin in green tea, has been identified for its anti-inflammatory activities, especially in the context of IBD [113]. EGCG has been shown to inhibit IFN-γ induced signalling pathways in intestinal epithelial cells and monocytes, thereby preventing the associated increase in epithelial permeability [97]. IFN-γ directly activates the transcription of the Th1-associated factor T-bet through the STAT 1 (STAT1) in CD4^+^ T cells [114]. However, EGCG administration has been observed to maintain the balance between Th1 and T-helper 2 (Th2) cells in UC models, potentially through modulation of the TLR4/MyD88/NF-κB signalling pathway [115].

*3.2.2.2.4*. *Genistein (Soybeans and Legumes).* Genistein (4,5,7-trihydroxyisoflavone) is a natural isoflavone compound recognised for its potent anti-inflammatory properties, making it a promising agent for preventing gastrointestinal diseases such as UC [116, 117]. Its therapeutic potential is largely attributed to its ability to modulate key inflammatory signalling pathways. Specifically, genistein has been shown to downregulate the IFN-γ/JAK1/STAT1 and IFN-γ/TLR-4/NF-κB signalling pathways, both of which contribute to gut inflammation by promoting pro-inflammatory cytokine production and immune activation [118]. By inhibiting NF-κB pathway activation, genistein further reduces IFN-γ expression, thereby disrupting the inflammatory cascade and potentially alleviating UC related symptoms [91, 119].

*3.2.2.2.5*. *Berberine (Berberis Species and Goldenseal).* Berberine, an isoquinoline alkaloid found in various medicinal plants, has long been recognised for its anti-inflammatory effects [120, 121]. Its therapeutic potential in UC is linked to the suppression of the IFN-γ signalling pathway, a key mediator in colonic immune-inflammatory responses [92]. In UC mouse models, BBR significantly downregulated IFN-γ and its downstream targets—IRF8, Ifit1, Ifit3 and IRF1 [92]. This effect is partly mediated by BBR's ability to activate AMPK, which supports immune regulation and cellular homeostasis [122].

*3.2.2.2.6*. *Andrographolide (Andrographis paniculata).* Andrographolide, a labdane diterpenoid derived from *Andrographis paniculata*, has demonstrated significant anti-inflammatory effects in various inflammatory disease models [93]. These effects are primarily attributed to the suppression of NF-κB and MAPK signalling pathways, coupled with the activation of nuclear factor E2-related factor 2 (Nrf2) signalling [123, 124]. Studies have shown that andrographolide, at different concentrations, can significantly reduce levels of IFN-γ along with other cytokines such as IL-23 and IL-17 in patients with UC, thereby diminishing the proportion of Th1 and Th17 helper T cells [125]. Additionally, andrographolide's anti-inflammatory properties are believed to be mediated through the downregulation of NF-κB, a key transcription factor that regulates inflammatory responses [126, 127].

*3.2.2.2.7*. *Luteolin (Celery, Green Peppers and Chamomile).* Luteolin, a flavonoid abundant in *Salvia tomentosa*, has demonstrated protective effects in colitis by attenuating colon shortening and reducing histological inflammation [128]. Nishitani et al. [94], reported that luteolin significantly suppressed the infiltration of macrophages and IFN-γ–producing CD4+ T cells into the colonic mucosa. Its anti-inflammatory action is mediated through inhibition of the JAK–STAT pathway, leading to decreased production of pro-inflammatory cytokines such as TNF-α and IL-6 [129]. These findings highlight luteolin's therapeutic potential in modulating immune responses in IBD.

*3.2.2.2.8*. *Ginsenosides (Panax).* Ginsenosides, the main active ingredients in ginseng, have shown promising anti-inflammatory and immunomodulatory effects [95]. Studies have demonstrated that certain ginsenosides, such as Rg1, Rd and Rh2, can effectively reduce the expression and activity of IFN-γ in IBD models [95, 130, 131]. Ginsenosides exert their anti-inflammatory effects by modulating various signalling pathways involved in inflammation, including NF-κB and p38MAPK, ultimately leading to reduced production of pro-inflammatory cytokines like IFN-γ [131, 132].

### 3.3. Comparative Efficacy and Safety of Biologics

Biologic therapies have transformed the management of IBD, but their efficacy and safety profiles vary considerably depending on the therapeutic target. Anti-TNF agents, such as infliximab and adalimumab, remain the most widely used biologics and have demonstrated robust efficacy in inducing and maintaining remission in moderate-to-severe IBD [133, 134]. However, limitations include primary non-response in up to one-third of patients, secondary loss of response and increased risk of infections due to systemic immunosuppression [135, 136]. Anti-IL-12/23 therapies including ustekinumab have emerged as effective alternatives, offering durable efficacy with a more favourable safety profile compared with anti-TNF therapy, particularly regarding immunogenicity and long-term tolerability [137–139]. In contrast, anti-IL-17A therapies, while useful for other autoimmune diseases, are generally not used for IBD because they can worsen the condition in clinical trials [140–143]. Direct IFN-γ blockade include fontolizumab and emapalumab has so far shown limited success. Fontolizumab demonstrated modest efficacy in CD but failed to achieve consistent clinical benefit, leading to discontinuation of its development [49]. Emapalumab, though effective in conditions characterised by hyper-inflammation, remains investigational in IBD and requires further evaluation to determine its therapeutic role [43]. Small-molecule JAK inhibitors, such as tofacitinib and upadacitinib, indirectly suppress IFN-γ along with multiple other cytokines and have demonstrated significant efficacy, especially in UC [144–146]. Their oral administration, rapid onset of action and steroid-sparing effects make them attractive options [147]. Nevertheless, risks such as thromboembolism and lipid abnormalities, requiring careful patient selection, risk factor assessment and close monitoring during IBD treatment [147]. Overall, anti-TNF agents offer strong efficacy but higher immunosuppressive risk, anti-IL-12/23 therapies combine durable response with better safety, anti-IL-17A agents are contraindicated and anti-IFN-γ therapies remain limited in clinical utility.

Compared with traditional therapies such as corticosteroids and immunomodulators, biologics and JAK inhibitors provide higher rates of mucosal healing, improved quality of life and long-term steroid-sparing benefits [148]. Looking forward, combination strategies—such as integrating cytokine inhibitors with microbiome-modulating interventions or conventional immunomodulators—may further optimise therapeutic outcomes while minimising adverse events, providing a comprehensive approach to IBD management.

## 4. Challenges and Future Directions

Despite the expanding body of evidence underscoring the pivotal role of IFN-γ in the pathogenesis of IBD, significant obstacles persist in the clinical translation of IFN-γ-targeted therapeutics. The multifactorial and heterogeneous nature of IBD, coupled with substantial inter-individual variability in therapeutic response, complicates the development of universally efficacious IFN-γ inhibitors. Moreover, the immunomodulatory nature of IFN-γ blockade raises concerns regarding heightened susceptibility to opportunistic infections and impaired host immune surveillance, which have led to the premature termination of several clinical trials due to adverse safety profiles. Compounding these challenges, the inherently low bioavailability and suboptimal pharmacokinetics of current IFN-γ antagonists further constrain their clinical utility.

Ongoing research endeavours continue to dissect the molecular intricacies of IFN-γ signalling in the context of intestinal inflammation, aiming to delineate the context-dependent effects of its downstream effectors. Particular emphasis is being placed on elucidating the immunopathological thresholds at which IFN-γ shifts from protective to deleterious, in order to design therapeutics that selectively modulate its activity. Central to this pursuit is the development of next-generation inhibitors with enhanced target specificity and efficacy, capable of attenuating pathogenic signalling without compromising essential immune functions.

In parallel, the refinement of IFN-γ-targeted strategies necessitates a paradigm shift towards precision medicine, incorporating robust patient stratification frameworks to identify individuals most likely to derive benefit. Emerging approaches in nanotechnology-mediated drug delivery offer promising avenues to improve tissue-specific targeting and enhance the local bioavailability of these agents, thereby reducing systemic toxicity. Additionally, the integration of high-resolution multi-omics platforms—such as transcriptomics, proteomics and single-cell sequencing—holds immense potential for the identification of predictive biomarkers and mechanistic disease subtypes.

Future therapeutic paradigms may also involve combinatorial regimens wherein IFN-γ inhibitors are co-administered with microbiome-modulating agents, stem cell-based interventions or other immunoregulatory modalities to synergistically restore intestinal homeostasis and achieve sustained remission. Ultimately, addressing these multifaceted challenges through interdisciplinary and translational research will be critical for unlocking the full therapeutic potential of IFN-γ inhibition in the long-term management of IBD.

## Figures and Tables

**Table 1:** Monoclonal antibodies targeting IFN-γ in inflammatory bowel disease.

Monoclonal antibody	Target	Mechanism of action	Study model	Key outcomes	Clinical status	References
Fontolizumab	IFN-γ	Neutralises circulating IFN-γ	Clinical (phase II)	Decreased disease severity and improved clinical response	Discontinued (lack of efficacy)	[40, 41]
Emapalumab	IFN-γ	Binds to IFN-γ and prevents receptor interaction, downregulating inflammatory pathways	Clinical (phase II)	Reduced IFN-γ-driven inflammation and improved mucosal healing	Investigational for IBD and currently approved for HLH	[42, 43]

**Table 2:** Indirect effects of JAK–STAT inhibitors on IFN-γ suppression in inflammatory bowel disease.

JAK–STAT inhibitor	Targeted pathway	Indirect mechanism of IFN-γ inhibition	Study model	Key outcomes	References
Tofacitinib	JAK1/JAK3	Reduces Th1 and Th17 differentiation, suppressing IFN-γ production	Clinical and preclinical	Improved mucosal healing and reduced inflammatory cytokines	[53]
Upadacitinib	JAK1	Preventing the phosphorylation of downstream effector proteins	Phase 3 clinical trials	Reduce inflammation in the gut	[54–56]
Filgotinib	JAK1	Reduces IFN-γ in serum	Phase 2 study	Improvements in clinical scores	[57, 58]
Ruxolitinib	JAK1 and JAK2	Disrupt the IFN-γ signalling pathway	In vivo study	Mitigating inflammation	[59–61]
Baricitinib	JAK2/STAT3	Impact T cell proliferation and differentiation	In vivo study	Help to reduce inflammation	[62]
Peficitinib	JAK1, JAK2, JAK3 and Tyk2	Inhibiting various inflammatory factors	Phase 2b trial	Mucosal healing, remission and clinical response in individuals receiving dosages greater than 75 mg o.d.	[63, 64]

**Table 3:** Plant compounds act as an IFN-γ inhibitor during IBD.

Compounds	Source	Study model	Dose/concentration	Mechanism	Key findings	Reference
Curcumin	*Curcuma longa* (turmeric)	In vivo	Concentration of 0.25% added in diet at 5 days before TNBS treatment	Suppresses IFN-γ expression via JAK–STAT pathway inhibition	Reduced colonic inflammation and improved gut barrier function	[88]
Resveratrol	*Japanese knotweed*	In vivo	75, 150 or 300 p.p.m.	Modulates CD3+ T cells that express interferon gamma	Suppress colitis and improve inflammation	[89]
Epigallocatechin gallate	Green tea	In vivo	10 μM	Blocked IFN-γ–triggered phosphorylation of JAK1, JAK2, and STAT3, and downregulated the expression of the pro-inflammatory gene CXCL9 in CD4+ T cells by preventing the formation of STAT1 homodimers	Regulating IFN-γ signalling and controlling inflammation	[90]
Genistein	Soybeans and legumes	In vitro and in vivo	0.1–2 and 0.5 mM	Inhibited the overexpression of IFN-γ	Alleviated colonic injury, inflammation and gut dysfunction	[91]
Berberine	Berberis species and goldenseal	In vitro and in vivo	20 and 40 mg/kg/d	IFN-γ and its initiated targets, including IRF8, Ifit1, Ifit3 and IRF1, were suppressed	Showing anti-inflammatory activity in the UC	[92]
Andrographolide	*Andrographis paniculata*	In vivo	50 and 100 mg/kg	Reduced proinflammatory cytokines by suppressing NF-κB, MAPK	Ameliorate DSS-induced acute colitis in mice	[93]
Luteolin	Celery, green pepper, perilla leaves and seeds and chamomile	In vivo	20 and 50 mg/kg	Suppressed the recruitment of macrophages and CD4+ T cells producing IFN-γ	Inhibits inflammation	[94]
Ginsenosides	Ginseng	In vivo	—	Reduce the expression and activity of IFN-γ	Showing anti-inflammatory effects	[95]

## Data Availability

The authors have nothing to report.
